# Supplementary material for: The structure of the Vibrio alginolyticus flagellar filament suggests molecular mechanism for the rotation of sheathed flagella
Source: Nat Commun. 2026 Apr 23;17:3532. doi: 10.1038/s41467-026-71203-7 (PMC13106801; doi:10.1038/s41467-026-71203-7)
Supplement: Supplementary file 1 — Supplementary Information [file 41467_2026_71203_MOESM1_ESM.pdf]

The structure of the *Vibrio alginolyticus* flagellar filament suggests molecular mechanism for the rotation of sheathed flagella

Kailin Qin[1,4*], Rosa Einenkel[2,4], Weilong Zhao[1], Joseph Atherton[1], Caroline Kühne[2], Marc Erhardt[2,3,*], Julien R. C. Bergeron[1,*]

Supplementary material:

Supplementary figures 1-9
Supplementary tables 1-5

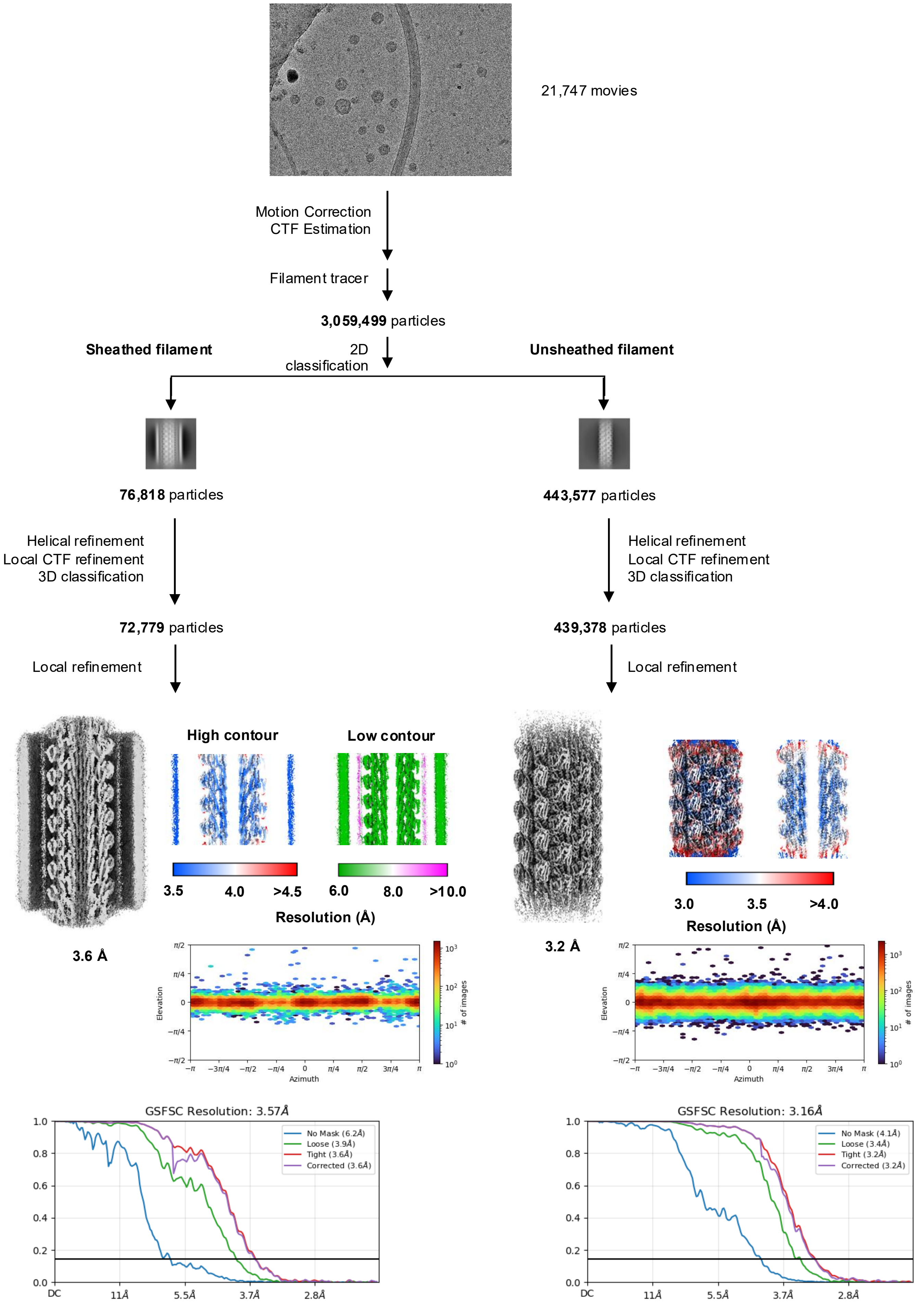

Figure S1. Cryo-EM data processing workflow of the sheathed and the unsheathed flagellar filament of *V. alginolyticus*. The various steps used for processing are shown, with local resolution, angular distribution, and FSC curves shown for both structures.

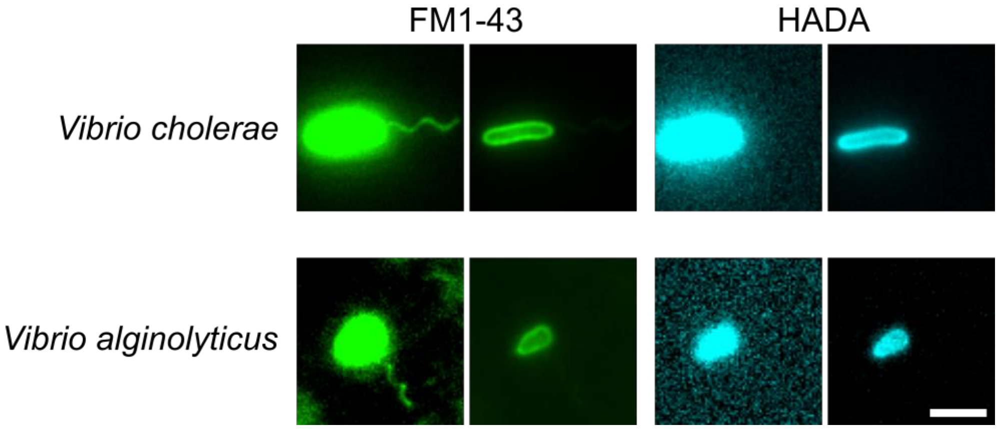

Supplementary Figure 2. The flagellar sheath does not contain peptidoglycan. Representative fluorescence microscopy images of *V. cholerae* and *V. alginolyticus* cells stained with FM1-43 (green) to visualise the outer membrane and flagellar sheath, and the fluorescent D-amino acid HADA (cyan) to label peptidoglycan. Scale bar, 3 µm.

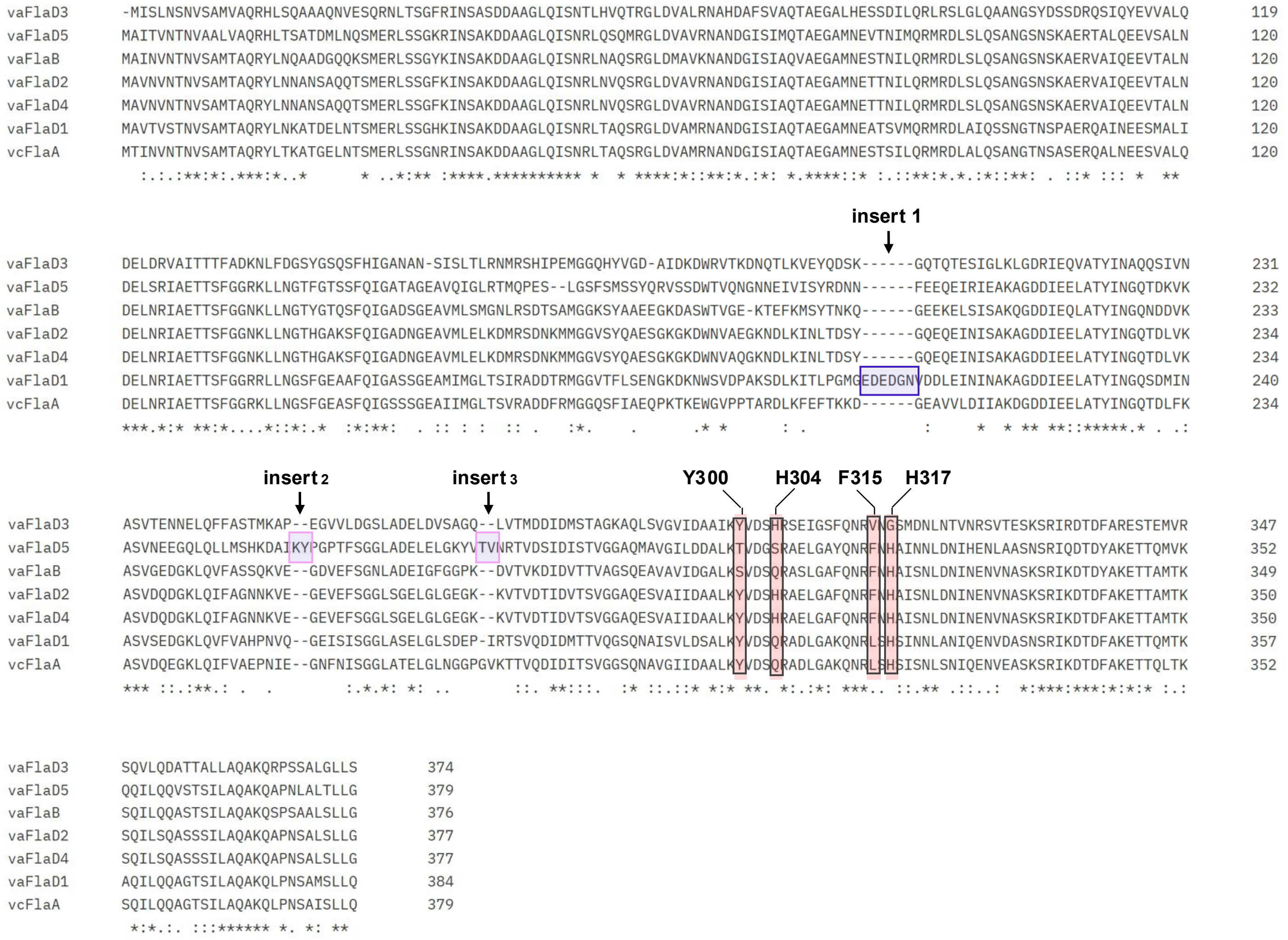

Supplementary Figure 3. Multiple sequence alignment of the *V. alginolyticus* flagellin sequences. The three inserts, and four residues, involved in identifying the correct flagellin in our filament structure are indicated.

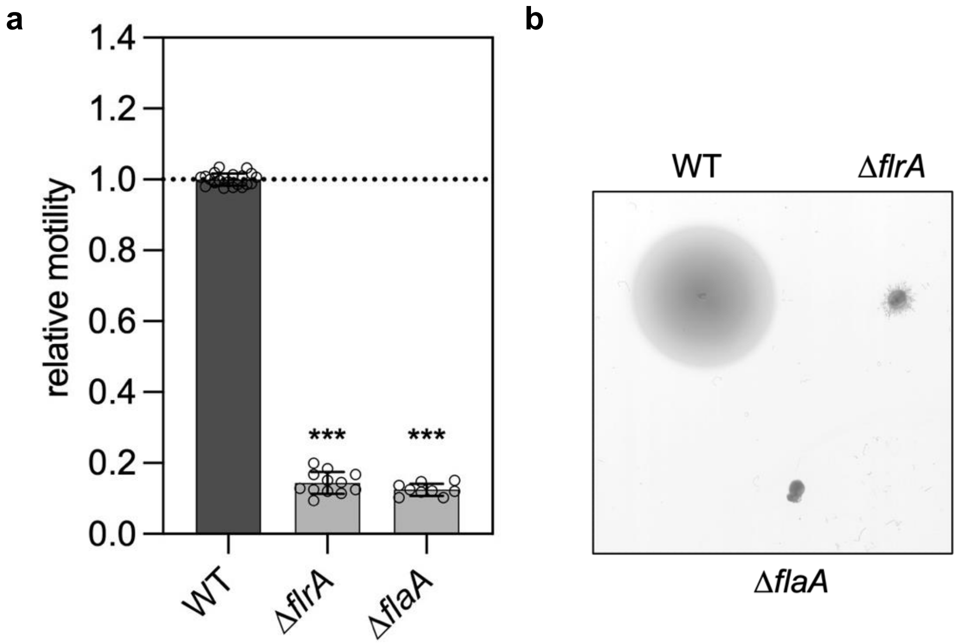

Supplementary Figure 4. FlaA is the main flagellin of the *V. cholerae* flagellar filament. a, Relative motility of *V. cholerae* Δ*flrA* (non-motile control) and Δ*flaA* (essential flagellin) mutants, analyzed using soft-agar motility plates after 6-8 h incubation at 37 °C. Motility halos were measured using Fiji and normalized to the WT. Bar graphs show mean ± SD from ≥9 biological replicates with individual data points. **b,** Representative swimming halos for the strains shown in a. Statistical analysis was performed using one-way ANOVA followed by Dunnett's multiple comparisons test (GraphPad Prism). *, $p < 0.05$; **, $p < 0.01$; ***, $p < 0.001$; ns, non-significant. Source data are provided as a Source Data file.

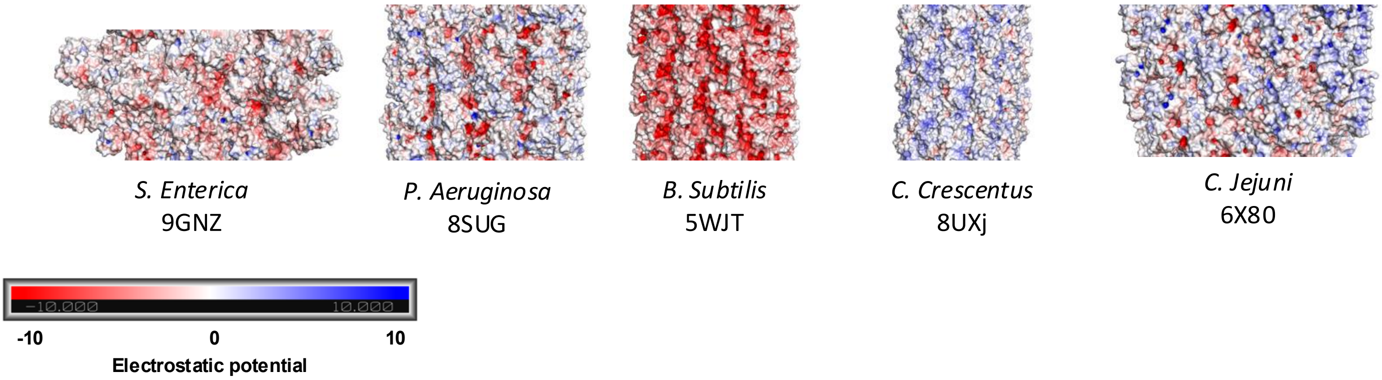

Supplementary Figure 5. Electrostatic surface of flagellar filaments across bacteria. A surface representation of the flagellum filament structure for divergent bacterial species are shown, colored by electrostatic potential (in kT/e). No obvious trend is observed, with most having a largely neutral surface, with the exception of the *B. subtilis* filament which is electronegative.

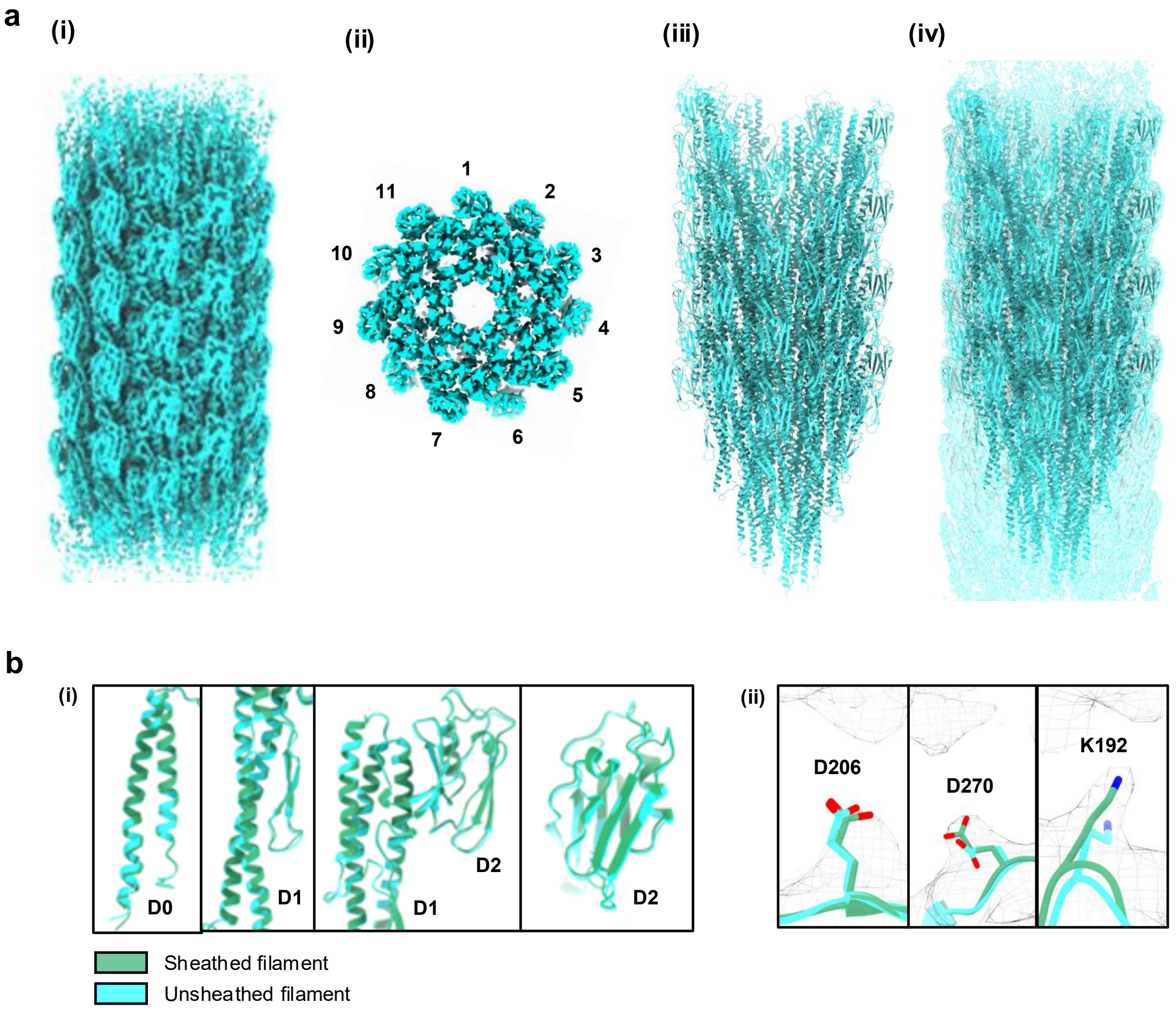

Supplementary Figure 6. Structure of unsheathed filaments from *V. alginolyticus*. a, Cryo-EM map of unsheathed filament from the side (i) and top (ii), and corresponding atomic model (iii) fitted into the map (iv). **b**, Comparison of the structure of the V. alginolyticus flagellum filament, with (green) and without (cyan) its sheath. No significant differences are identified, at the backbone level, for any of the domains (i); very minor differences are found to the side-chain of residues facing the membrane (ii).

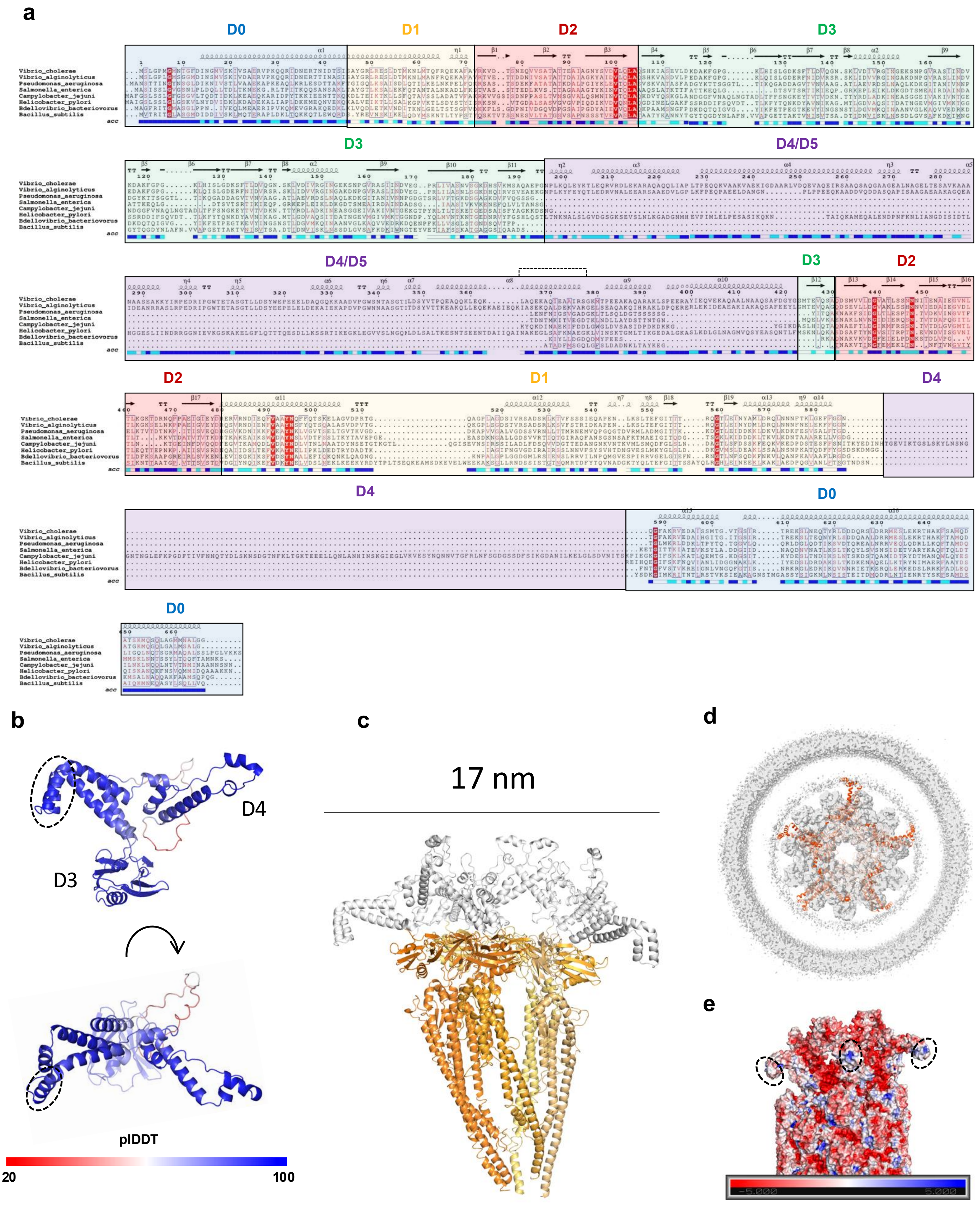

Supplementary Figure 7. Modeling of the *V. alginolyticus* cap complex. **a**, Multiple sequence alignment for FliD from different species. The domains are colour coded as follows: Blue: D0 terminal domains. Yellow: D1. Red: D2 domain. Green: D3 domain. Purple: D3 domain. **b**, Alphafold prediction of the D3 and D4 domain of FliD of *V. alginolyticus* colored according to the pIDDT score. **c**, Dimensions of the predicted FliD D4 domain structure of *V. alginolyticus*. **d**, Overlay of the cross-view 3D reconstruction of the sheathed filament (grey) and the top-view of the FliD D4 domain model (orange) of *V. alginolyticus.* The diameter of the D4 domain of FliD and the inner leaflet of the sheath are similar. **e**, Electrostatic surface representation for the *V. alginolyticus* filament-cap model. The Region of FliD D4 that is in close proximity to the sheath (dotted circle) is electropositive, unlike the surface of the filament, supporting a direct interaction with the membrane.

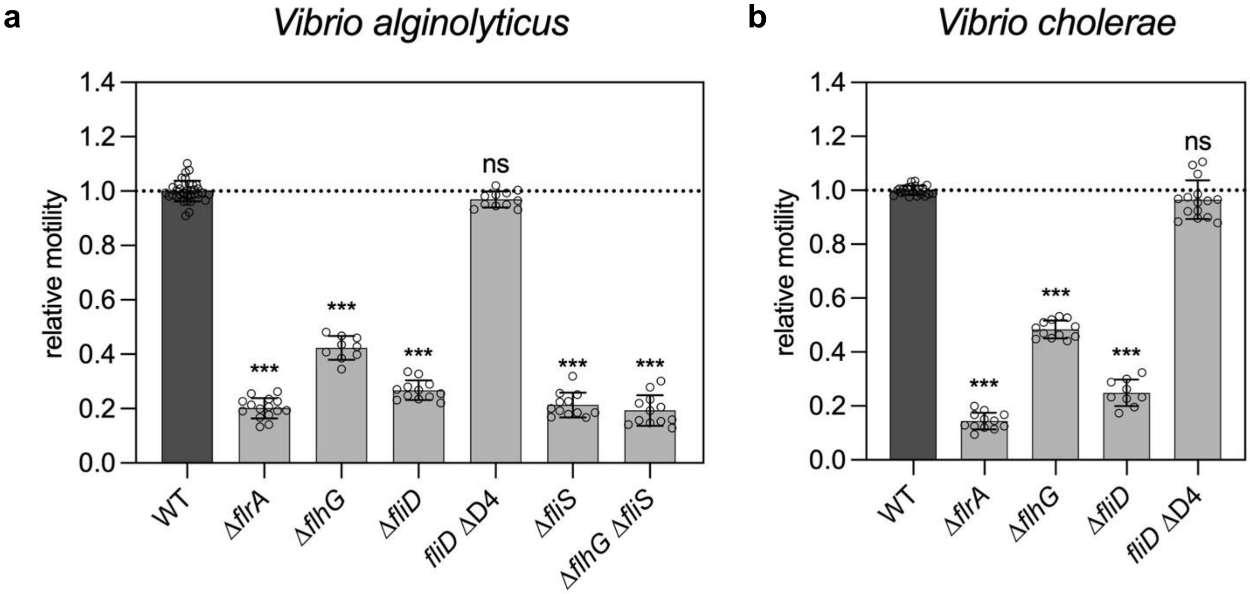

Supplementary Figure 8. Motility assays for the various mutants employed to characterize flagellum assembly. **a**, Relative motility of *V. alginolyticus* mutants, analyzed using soft-agar motility plates after 6-8 h incubation at 37 °C. **b**, Relative motility of *V. cholerae* mutants, analyzed using soft-agar motility plates after 6-8 h incubation at 37 °C. Motility halos were measured using Fiji and normalized to the WT. Bar graphs show mean ± SD from ≥9 biological replicates with individual data points. Statistical analysis was performed using one-way ANOVA followed by Dunnett's multiple comparisons test (GraphPad Prism). *, $p < 0.05$; **, $p < 0.01$; ***, $p < 0.001$; ns, non-significant. Source data are provided as a Source Data file.

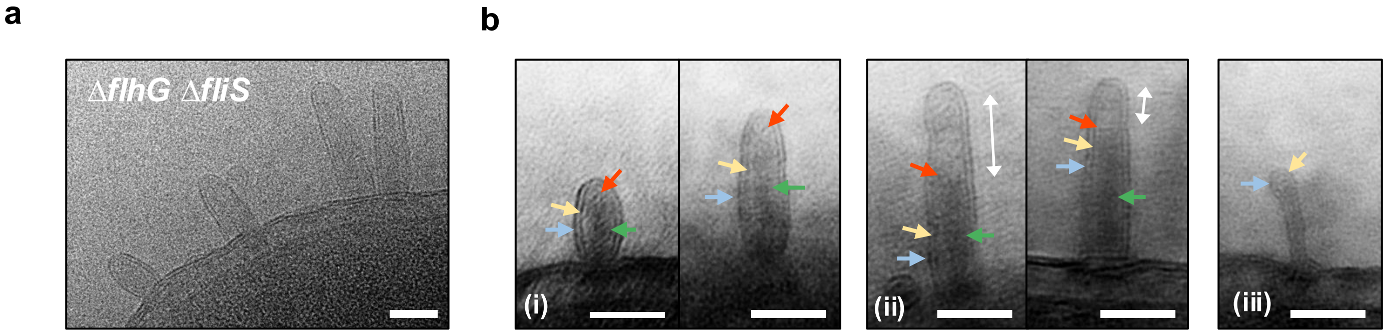

Supplementary Figure 9. Position of cap complexes at early stages of filament assembly. a, Slice of a representative cryo-electron tomograph of a hyperflagellated *V. alginolyticus* strain with impaired flagellin secretion (Δ*flhG* Δ*fliS*). **b**, close-up views showing distinct flagellar architectures in which the (i) cap complex is located near the sheath, the (ii) filament is shorter and the cap is located below the sheath and (iii) empty sheath without a filament. Arrows indicate the membrane outer layer (blue), inner layer (yellow), filament (green) and cap (orange).

Supplementary table 1: Cryo-EM data collection, refinement and validation statistics

	**#1 Sheathed filament (EMD-53917) (PDB 9RCD)**	**#2 Unsheathed filament (EMD-53912) (PDB 9RCB)**
**Data collection and processing**		
Camera	K3	
Magnification	81,000	
Voltage (kV)	300	
Electron exposure (e^{-}/ $Å^2$)	40	
Defocus range (μm)	-0.9 to -2.7	
Pixel size (Å)	1.1	
Symmetry imposed	C1	C1
Initial particle images (no.)	76,818	443,577
Final particle images (no.)	72,779	439,378
FSC threshold	0.143	0.143
Map resolution (Å)	3.6	3.16
**Refinement**		
Initial model used (PDB code)	AlphaFold DB	AlphaFold DB
Map-to-Model resolution (Å)		
Masked	4.3	3.2
Unmasked	4.7	3.3
Model Vs Data CC		
Mask	0.76	0.75
Box	0.56	0.63
Local resolution range (Å)	3.6 to 10.0	3.2 to 4.5
Map sharpening B factor ($Å^2$)	-17.5	-73.5
Number of chains	33	55
Model composition<br>Non-hydrogen atoms<br>Protein residues	123112<br>16544	154330<br>20735
B factors ($Å^2$)<br>Protein	159.3	134.25
R.m.s. deviations<br>Bond lengths (Å)<br>Bond angles (°)	0.006<br>1.033	0.006<br>0.917
Validation<br>MolProbity score<br>Clashscore<br>Poor rotamers (%)	1.94<br>18.69<br>1.48	1.84<br>15.89<br>1.52
Ramachandran plot<br>Favored (%)<br>Allowed (%)<br>Disallowed (%)	97.80<br>2.18<br>0.02	98.60<br>1.40<br>0.00

Supplementary table 2: The sequence identity matrix of flagellin homologs in *Vibrio alginolyticus*

	FlaD3	FlaD5	FlaD1	FlaB	FlaD2	FlaD4
FlaD3	100.0	47.3	44.4	50.9	52.1	52.1
FlaD5	47.3	100.0	60.9	65.2	67.2	67.2
FlaD1	44.4	60.9	100.0	64.9	66.8	66.8
FlaB	50.9	65.2	64.9	100.0	78.5	78.2
FlaD2	52.1	67.2	66.8	78.5	100.0	99.7
FlaD4	52.1	67.2	66.8	78.2	99.7	100.0

Supplementary table 3: Bacterial strains used in this study

Strain ID	Genotype	Source/Reference
Vibrio alginolyticus NCTC 10675		
EM18481	WT, Amp^R	Sakazaki et al., 1963
EM18568	Δ*flrA*	This study
EM18570	Δ*flhG*	This study
EM18833	Δ*fliS*	This study
EM18834	Δ*flhG* Δ*fliS*	This study
EM18835	*fliD* ΔD4 (Δaa206-418)	This study
EM18896	Δ*fliD*	This study
EM19615	Δ*lafK*	This study
EM19616	Δ*flrA* Δ*lafK*	This study
EM19617	Δ*flaD2* Δ*lafK*	This study
EM19678	Δ*flaB* Δ*lafK*	This study
EM19679	Δ*flaD1* Δ*lafK*	This study
EM19680	Δ*flaD3* Δ*lafK*	This study
EM19681	Δ*flaD4* Δ*lafK*	This study
EM19682	Δ*flaD5* Δ*lafK*	This study
Vibrio cholerae V52		
EM16870	Δ*vasK* (T6SS negative), $Strep^R$	Pukatzki et al., 2006
EM18273	Δ*vasK* Δ*flrA*	This study
EM18356	Δ*vasK* Δ*flhG*	This study
EM18564	Δ*vasK* *fliD* ΔD4 (Δaa207-418)	This study
EM18565	Δ*vasK* Δ*flaA*	This study
EM18897	Δ*vasK* Δ*fliD*	This study
Escherichia coli		
TH12219	CC118 λ-pir^+	Lab collection
EM18188	CC118 λ-pir^+ / pEM18188 (pRE112-Δ*flrA* (from *Vibrio cholerae*), Cm^R)	This study
EM18320	CC118 λ-pir^+ / pEM18320 (pRE112-Δ*flhG* (from *Vibrio cholerae*), Cm^R)	This study
TH6708	DH5α λ-pir^+	Lab collection
EM18524	DH5α λ-pir^+ / pEM18524 (pRE112-Δ*flrA* (from *Vibrio alginolyticus*), Cm^R)	This study
EM18541	DH5α λ-pir^+ / pEM18541 (pRE112-Δ*flaA* (from *Vibrio cholerae*), Cm^R)	This study
EM18542	DH5α λ-pir^+ / pEM18542 (pRE112-*fliD* ΔD4 (from *Vibrio cholerae*), Cm^R)	This study
EM18543	DH5α λ-pir^+ / pEM18543 (pRE112-Δ*flaB* (from *Vibrio alginolyticus*), Cm^R)	This study
EM18544	DH5α λ-pir^+ / pEM18544 (pRE112-Δ*flhG* (from *Vibrio alginolyticus*), Cm^R)	This study
EM18734	DH5α λ-pir^+ / pEM18734 (pRE112-Δ*fliS* (from *Vibrio alginolyticus*), Cm^R)	This study
EM18735	DH5α λ-pir^+ / pEM18735 (pRE112-*fliD* ΔD4 (from *Vibrio alginolyticus*), Cm^R)	This study
EM18840	DH5α λ-pir^+ / pEM18840 (pRE112-Δ*fliD* (from *Vibrio cholerae*), Cm^R)	This study
EM18841	DH5α λ-pir^+ / pEM18841 (pRE112-Δ*fliD* (from *Vibrio alginolyticus*), Cm^R)	This study
EM19125	DH5α λ-pir^+ / pEM19125 (pRE112-Δ*flaD1* (from *Vibrio alginolyticus*), Cm^R)	This study
EM19126	DH5α λ-pir^+ / pEM19126 (pRE112-Δ*flaD2* (from *Vibrio alginolyticus*), Cm^R)	This study
EM19127	DH5α λ-pir^+ / pEM19127 (pRE112-Δ*flaD3* (from *Vibrio alginolyticus*), Cm^R)	This study
EM19128	DH5α λ-pir^+ / pEM19128 (pRE112-Δ*flaD4* (from *Vibrio alginolyticus*), Cm^R)	This study
EM19129	DH5α λ-pir^+ / pEM19129 (pRE112-Δ*flaD5* (from *Vibrio alginolyticus*), Cm^R)	This study
EM19517	DH5α λ-pir^+ / pEM19517 (pRE112-Δ*lafK* (from *Vibrio alginolyticus*), Cm^R)	This study
TH4042	SM10 λ-pir^+	Lab collection
EM18154	SM10 λ-pir^+ / pEM18188 (pRE112-Δ*flrA* (from *Vibrio cholerae*), Cm^R)	This study
EM18322	SM10 λ-pir^+ / pEM18320 (pRE112-Δ*flhG* (from *Vibrio cholerae*), Cm^R)	This study
EM18534	SM10 λ-pir^+ / pEM18524 (pRE112-Δ*flrA* (from *Vibrio alginolyticus*), Cm^R)	This study
EM18546	SM10λ-pir^+ / pEM18541 (pRE112-Δ*flaA* (from *Vibrio cholerae*), Cm^R)	This study
EM18547	SM10 λ-pir^+ / pEM18542 (pRE112-*fliD* ΔD4 (from *Vibrio cholerae*), Cm^R)	This study
EM18548	SM10 λ-pir^+ / pEM18543 (pRE112-Δ*flaB* (from *Vibrio alginolyticus*), Cm^R)	This study
EM18549	SM10 λ-pir^+ / pEM18544 (pRE112-Δ*flhG* (from *Vibrio alginolyticus*), Cm^R)	This study
EM18738	SM10 λ-pir^+ / pEM18734 (pRE112-Δ*fliS* (from *Vibrio alginolyticus*), Cm^R)	This study
EM18739	SM10 λ-pir^+ / pEM18735 (pRE112-*fliD* ΔD4 (from *Vibrio alginolyticus*), Cm^R)	This study
EM18842	SM10 λ-pir^+ / pEM18840 (pRE112-Δ*fliD* (from *Vibrio cholerae*), Cm^R)	This study
EM18843	SM10 λ-pir^+ / pEM18841 (pRE112-Δ*fliD* (from *Vibrio alginolyticus*), Cm^R)	This study
EM19130	SM10 λ-pir^+ / pEM19125 (pRE112-Δ*flaD1* (from *Vibrio alginolyticus*), Cm^R)	This study
EM19131	SM10 λ-pir^+ / pEM19126 (pRE112-Δ*flaD2* (from *Vibrio alginolyticus*), Cm^R)	This study
EM19132	SM10 λ-pir^+ / pEM19127 (pRE112-Δ*flaD3* (from *Vibrio alginolyticus*), Cm^R)	This study
EM19133	SM10 λ-pir^+ / pEM19128 (pRE112-Δ*flaD4* (from *Vibrio alginolyticus*), Cm^R)	This study
EM19134	SM10 λ-pir^+ / pEM19129 (pRE112-Δ*flaD5* (from *Vibrio alginolyticus*), Cm^R)	This study
EM19524	SM10 λ-pir^+ / pEM19517 (pRE112-Δ*lafK* (from *Vibrio alginolyticus*), Cm^R)	This study

Supplementary table 4: List of plasmids used in this study

Plasmid	Description	Source/Reference
pRE112	pGP704, *sacB*, Cm^R	Lab collection
pEM18188	pRE112-Δ*flrA* (from *Vibrio cholerae*), Cm^R	This study
pEM18320	pRE112-Δ*flhG* (from *Vibrio cholerae*), Cm^R	This study
pEM18524	pRE112-Δ*flrA* (from *Vibrio alginolyticus*), Cm^R	This study
pEM18541	pRE112-Δ*flaA* (from *Vibrio cholerae*), Cm^R	This study
pEM18542	pRE112-*fliD* ΔD4 (from *Vibrio cholerae*), Cm^R	This study
pEM18543	pRE112-Δ*flaB* (from *Vibrio alginolyticus*), Cm^R	This study
pEM18544	pRE112-Δ*flhG* (from *Vibrio alginolyticus*), Cm^R	This study
pEM18734	pRE112-Δ*fliS* (from *Vibrio alginolyticus*), Cm^R	This study
pEM18735	pRE112-*fliD* ΔD4 (from *Vibrio alginolyticus*), Cm^R	This study
pEM18840	pRE112-Δ*fliD* (from *Vibrio cholerae*), Cm^R	This study
pEM18841	pRE112-Δ*fliD* (from *Vibrio alginolyticus)*, Cm^R	This study
pEM19125	pRE112-Δ*flaD1* (from *Vibrio alginolyticus)*, Cm^R	This study
pEM19126	pRE112-Δ*flaD2* (from *Vibrio alginolyticus)*, Cm^R	This study
pEM19127	pRE112-Δ*flaD3* (from *Vibrio alginolyticus)*, Cm^R	This study
pEM19128	pRE112-Δ*flaD4* (from *Vibrio alginolyticus*), Cm^R	This study
pEM19129	pRE112-Δ*flaD5* (from *Vibrio alginolyticus*), Cm^R	This study
pEM19517	pRE112-Δ*lafK* (from *Vibrio alginolyticus*), Cm^R	This study

Supplementary table 5: List of oligonucleotides used in this study

Primer Name	Sequence	Source
CAT-C1	TTATACGCAAGGCGACAAGG	Lab Collection
pRE112 seq fw	GATTTTCTGGTGCGTACCGG	This study
pRE112 linearize fw	CTCTAGAAGAAGCTTGGGATCG	This study
pRE112 linearize rev	AGCTCTCCCGGGAATTCA	This study
DflrA clean fw1_VIBCH	GTGTAAGTGAACTGCATGAATTCCCGGGAGAGCTGATATCATCATTAGCCTAC	This study
DflrA clean rev1_VIBCH	CCCATCCACTATAAACTAACACGCTAAACTCTGCATAGGT	This study
DflrA clean fw2_VIBCH	TGTTAGTTTATAGTGGATGGGGAAGAGCCGCAGGAGTTTG	This study
DflrA clean rev2_VIBCH	GTGATAGGGCCCGATCCCAAGCTTCTTCTAGAGCTTCTGGGTTGGCTTCACG	This study
DflrA colony PCR fw_VIBCH	GTAAGGCGGCAATGCAGC	This study
DflrA colony PCR rev_VIBCH	GCAATCTGGATCACGCTG	This study
pRE112-DflrA up fw_VIBAL	CATGAATTCCCGGGAGAGCTGGTTAAGCAGGATGCAGAGTG	This study
DflrA up rev_VIBAL	CGCATTTTTTCAATTACAAGCAGCTTTGCCAAAC	This study
DflrA down fw_VIBAL	GCTGCTTGTAATTGAAAAAATGCGTAAGTACAAC	This study
pRE112-DflrA down rev_VIBAL	CGATCCCAAGCTTCTTCTAGAGCTCAGTTTGTTCTTCTTC	This study
DflrA colony fw_VIBAL	GGTCGATATCAGGGAACGGTTATTGC	This study
DflrA colony rev_VIBAL	CGGATGCAATGGCGTTCGC	This study
DflhG pRE112 fw_VIBCH	GTGTAAGTGAACTGCATGAATTCCCGGGAGAGCTGATGTCCTGTAAGAGTTG	This study
DflhG upstream rev_VIBCH	CCCATCCACTATAAACTAACATTGGTCATATATCATTTTGTTTG	This study
DflhG downstream fw_VIBCH	TGTTAGTTTATAGTGGATGGGCGGTCAGAAACAGTAGGGGAAC	This study
DflhG pRE112 rev_VIBCH	GTGATAGGGCCCGATCCCAAGCTTCTTCTAGAGCACCGAGATCCTCTATCC	This study
DflhG colony PCR fw_VIBCH	CGTCGCATTGGTGACCAC	This study
DflhG colony PCR rev_VIBCH	CAACGAGTGCTTTACGGAAAG	This study
DflhG down rev_VIBAL	CGATCCCAAGCTTCTTCTAGAGATCCGCCGGTGAAATCAC	This study
DflhG down fw_VIBAL	AATCGTACCGAATTCGCAGAG	This study
DflhG up rev_VIBAL	CTCTGCGAATTCGGTACGATTTGCTTGATCGTGTATCATATTCTC	This study
DflhG up fw_VIBAL	TGAATTCCCGGGAGAGCTGTTGTCCCGTAAGAGTTGCT	This study
DflhG colony fw_VIBAL	CCCCTTTGAATGGGTTGCTG	This study
DflhG colony rev_VIBAL	GGTGCACATGAGCAACTCTC	This study
DflaA up fw_VIBCH	GAACTGCATGAATTCCCGGGAGAGCTCGAAGATTTCAAAATCAGC	This study
DflaA up rev_VIBCH	GAGTTTGGCAACGACACGTTGGTATTTACG	This study
DflaA down fw_VIBCH	GTAAATACCAACGTGTCGTTGCCAAACTCTGCAATC	This study
DflaA down rev_VIBCH	GGCCCGATCCCAAGCTTCTTCTAGAGCCATAGTGGTATCTCCTATTG	This study
DflaA fw_VIBCH	CAAACTGCATCAGTTAGTTGAAG	This study
DflaA rev_VIBCH	GTTGTGCTGTCATTGCTGAC	This study
DflaB up fw_VIBAL	GAACTGCATGAATTCCCGGGAGAGCTCGAAGGCAAAAAAGTAACG	This study
DflaB up rev_VIBAL	GCGCTGCAGATGGAGAAGAAACGTTAGTATTAACGTTAATC	This study
DflaB down fw_ViBAL	CTAACGTTTCTTCTCCATCTGCAGCGCTAAGCTTG	This study
DflaB down rev_ViBAL	GGCCCGATCCCAAGCTTCTTCTAGAGCCATGCCGCCAGACATCCC	This study
DflaB fw_VIBAL	GGGCGAGGTCGAGTTTTCAGG	This study
DflaB rev_VIBAL	GTACGCGTTCGGCATCCAC	This study
fliD DD4 up fw_VIBCH	GAACTGCATGAATTCCCGGGAGAGCTGGTCAGCAAAATTGTCAGTG	This study
fliD DD4 up rev_VIBCH	CGTAGCCATCGAGGGTTTTGTATTCGAGTTG	This study
fliD DD4 down fw_VIBCH	CTCGAATACAAAACCCTCGATGGCTACGGCGGCATG	This study
fliD DD4 down rev_VIBCH	GGCCCGATCCCAAGCTTCTTCTAGAGGTCTGTTCATTCAAGCTC	This study
fliD DD4 fw_VIBCH	GAGGTGATTGAATGAGTTTAGG	This study
fliD DD4 rev_VIBCH	GGGTCCGTTTTTCCAAAC	This study
pRE-DfliS down rev_VIBAL	GTGATAGGGCCCGATCCCAAGCTTCTTCTAGAGCAGGCAAAGGAAAACGGC	This study
pRE-DfliS down fw_VIBAL	GCATACAAAAAGCATAATCTTACCGCTGCTGAAG	This study
pRE-DfliS up rev_VIBAL	GCGGTAAGATTATGCTTTTTGTATGCTTGCAAAGAAC	This study
pRE-DfliS up fw_VIBAL	GGTGTAAGTGAACTGCATGAATTCCCGGGAGAGCTCCAGTTGAACAATAACTTCAAC	This study
DfliS colony fw_VIBAL	GGCAGCTCTAGTTCACCAAC	This study
DfliS colony rev_VIBAL	CACGTCAAGGCACCCTAG	This study
fliD DD4 up fw_VIBAL	GGTGTAAGTGAACTGCATGAATTCCCGGGAGAGCTCAACAGCGGGTTGTTGAC	This study
fliD DD4 up rev_VIBAL	CCCAAGGTATTCTGTTTGGTATTCGAAATATTTAAGAGGG	This study
fliD DD4 down fw_VIBAL	CGAATACCAAACAGAATACCTTGGGATGACAGAAG	This study
fliD DD4 down rev_VIBAL	GTGATAGGGCCCGATCCCAAGCTTCTTCTAGAGCGTCACTCAAACGGTAGTTTTGTTC	This study
fliD DD4 fw_VIBAL	CTTTCCATGCGGCGATCTAATG	This study
fliD DD4 rev_VIBAL	GGTATTGCGTCGCCTAGC	This study
DfliD down rev_VIBCH	GTGATAGGGCCCGATCCCAAGCTTCTTCTAGAGGATTTTTGGCGATGTCGCCAC	This study
DfliD down fw_VIBCH	GATGAATACTTTAGCGGGCATGATGAACGC	This study
DfliD up rev_VIBCH	CATCATGCCCGCTAAAGTATTCATCCCCATCGGG	This study
DfliD up fw_VIBCH	GTGTAAGTGAACTGCATGAATTCCCGGGAGAGCTCTGAGCTTATTGGGATAACCC	This study
DfliD colony fw_VIBCH	GTTGACCATAAAGTCATACAG	This study
DfliD colony rev_VIBCH	GACGAAGGCGCAAATATTGC	This study
DfliD down rev_VIBAL	GTGATAGGGCCCGATCCCAAGCTTCTTCTAGAGGTTTGAAGCAATATCACCACCGTCATC	This study
DfliD down fw_VIBAL	GATGTCTGGCCAGTTGGGCGCTTTGATG	This study
DfliD up rev_VIBAL	CAAAGCGCCCAACTGGCCAGACATCCCCAAAGG	This study
DfliD up fw_VIBAL	GTGTAAGTGAACTGCATGAATTCCCGGGAGAGCTCTAAGCTTGTTGGGCTAAGTGG	This study
DfliD colony fw_VIBAL	GCGTGATCATAAACTCATAC	This study
DfliD colony rev_VIBAL	CTTCAGCAAGCAAGTACTTC	This study
DflaD_1 up fw_VIBAL	GGTGTAAGTGAACTGCATGAATTCCCGGGAGAGCTGTCTCTGGCAAAAGCGAAG	This study
DflaD_1 up rev_VIBAL	AGTTTGGCAAGGAGACGTTAGTACTAACTGTTACAG	This study
DflaD_1 down fw_VIBAL	TAACGTCTCCTTGCCAAACTCTGCAATGTC	This study
DflaD_1 down rev_VIBAL	GTGATAGGGCCCGATCCCAAGCTTCTTCTAGAGCATCTTTTGCGCTGTTGATTTTG	This study
DflaD_1 colony fw_VIBAL	CAAGAGCAAAATCATGAAGAC	This study
DflaD_1 colony rev_VIBAL	CGCATACGTTGAAGAATG	This study
DflaD_2 up fw_VIBAL	GGTGTAAGTGAACTGCATGAATTCCCGGGAGAGCTGCCAAACTCTGCAATGTCAC	This study
DflaD_2 up rev_VIBAL	AGTTAGGCGCTGATACGTTAGTGTTTACATTCACTG	This study
DflaD_2 down fw_VIBAL	CTAACGTATCAGCGCCTAACTCAGCGCTAAG	This study
DflaD_2 down rev_VIBAL	AAGTGATAGGGCCCGATCCCAAGCTTCTTCTAGAGGATTGAATTAGCGTTCGCGCC	This study
DflaD_2 colony fw_VIBAL	CATCGATTCTTGCTCAAGCG	This study
DflaD_2 colony rev_VIBAL	GATATGGCTACGCATGTTACG	This study
DflaD_3 up fw_VIBAL	GGTGTAAGTGAACTGCATGAATTCCCGGGAGAGCTCAAACTGACCTAGTGAAAGCG	This study
DflaD_3 up rev_VIBAL	GAACTCGGCCTCGCAGAAACATTGGAATTTAAAGAG	This study
DflaD_3 down fw_VIBAL	CAATGTTTCTGCGAGGCCGAGTTCGGCACTTG	This study
DflaD_3 down rev_VIBAL	AAGTGATAGGGCCCGATCCCAAGCTTCTTCTAGAGCAGCGCTGACGCTGGTGC	This study
DflaD_3 colony fw_VIBAL	CGAAGAGCTTGCGACTTAC	This study
DflaD_3 colony rev_VIBAL	GGGTCTGTTTGACAAACTTAAGAAG	This study
DflaD_4 up fw_VIBAL	GGTGTAAGTGAACTGCATGAATTCCCGGGAGAGCTGATATTAGCACGGTCGGTGGC	This study
DflaD_4 up rev_VIBAL	GAGTTTGGCGCTGATACGTTAGTGTTTACATTCACTG	This study
DflaD_4 down fw_VIBAL	CACTAACGTATCAGCGCCAAACTCAGCGCTAAG	This study
DflaD_4 down rev_VIBAL	AAGTGATAGGGCCCGATCCCAAGCTTCTTCTAGAGCGAAAGATCACGCATACGTTGTAGG	This study
DflaD_4 colony fw_VIBAL	GAGTTAGAGCCGTTCGCC	This study
DflaD_4 colony rev_VIBAL	CTTGGCAAGTATGTCACCG	This study
DflaD_5 up fw_VIBAL	GGTGTAAGTGAACTGCATGAATTCCCGGGAGAGCTGGCTCTAGTAAAAGAGGGCC	This study
DflaD_5 up rev_VIBAL	GGTTTGGCGCTGCAACATTGGTATTAACGG	This study
DflaD_5 down fw_VIBAL	CCAATGTTGCAGCGCCAAACCTAGCATTGAC	This study
DflaD_5 down rev_VIBAL	AAGTGATAGGGCCCGATCCCAAGCTTCTTCTAGAGCCGTTTGCTGATTGCAGAGACAAG	This study
DflaD_5 colony fw_VIBAL	CACTGCTTCGCCGTTATCC	This study
DflaD_5 colony rev_VIBAL	GGCACAGAAAACCCGAAAGG	This study
DlafK up fw VIBAL	GGTGTAAGTGAACTGCATGAATTCCCGGGAGAGCTGGATTCAATTGCTTCGGGTAG	This study
DlafK up rev	CCGAGCCTAGTTTCGTTTTCGTCATCATTGAACC	This study
DlafK down fw VIBAL	GATGACGAAAACGAAACTAGGCTCGGCTGCCTAATAG	This study
DlafK down rev VIBAL	GAATAAGTGATAGGGCCCGATCCCAAGCTTCTTCTAGAGGAGGAACTCCATAGTGCAACC	This study
DlafK colony fw VIBAL	CAAAACGCTCTTGTTGGCTATAG	This study
DlafK colony rev VIBAL	GAATTTACTAAGCCGTCTCAGCG	This study
